# Seasoning

**Published:** 1844-10-05

**Authors:** 


### SEASONING.

Dr. Armstrong says the influence of warm climates is apparent after a few years' residence within the tropics. Europeans lose their sanguineous complexions, and acquire the power of resisting the heat better than the new comer. This power of accommodation to circumstances arises from a corresponding change in the functions of life, and which is usually attributed to the individual having undergone the process of seasoning.—*Lond. Med. Times.*

